# The effect of ambient humidity on the electrical properties of graphene oxide films

**DOI:** 10.1186/1556-276X-7-363

**Published:** 2012-07-02

**Authors:** Yao Yao, Xiangdong Chen, Jinfeng Zhu, Baoqing Zeng, Zuquan Wu, Xiaoyu Li

**Affiliations:** 1 School of Information Science and Technology, Southwest Jiaotong University, Chengdu, 610031, People's Republic of China; 2 National Key Laboratory of Science and Technology on Vacuum Electronics, School of Physical Electronics, University of Electronic Science and Technology of China, Chengdu, 610054, People's Republic of China

**Keywords:** Graphene oxide, Humidity sensing, Complex impedance spectroscopy, Nano device

## Abstract

We investigate the effect of water adsorption on the electrical properties of graphene oxide (GO) films using the direct current (DC) measurement and alternating current (AC) complex impedance spectroscopy. GO suspension synthesized by a modified Hummer's method is deposited on Au interdigitated electrodes. The strong electrical interaction of water molecules with GO films was observed through electrical characterizations. The DC measurement results show that the electrical properties of GO films are humidity- and applied voltage amplitude-dependent. The AC complex impedance spectroscopy method is used to analyze the mechanism of electrical interaction between water molecules and GO films in detail. At low humidity, GO films exhibit poor conductivity and can be seen as an insulator. However, at high humidity, the conductivity of GO films increases due to the enhancement of ion conduction. Our systematic research on this effect provides the fundamental supports for the development of graphene devices originating from solution-processed graphene oxide.

## Background

Graphene oxide (GO), a single thin sheet of graphite oxide that conventionally serves as a precursor material for preparing graphene [1-3], has received increasing attentions in the application of optoelectronic [4,5] and sensor devices [6] due to its inherent electrical and mechanical properties. As an oxidation product of graphene, GO can be viewed as a two-dimension network of *sp*^2^ and *sp*^3^-bonded hybridized carbon atoms arranged in a dense honeycomb crystal structure. Many oxygen-containing groups, including hydroxyl, epoxy and carboxylic acid, were bonded to the two-dimension network. The presence of *sp*^3^-bonded hybridized carbon atoms weakens the conductivity and enhances the hydrophilic property of GO. Recently, exploring the feasibility of integrating GO into graphene-based electronic devices has motivated immense studies on the intrinsic electrical [7,8] and mechanical [9,10] properties of GO. It is well known that the electrical properties of GO would be influenced by some external stimulations, including reducibility reagent [11], electric field [12-14], temperature [15,16], light [17], etc. Due to the tunable electrical property, GO is considered as a potential electrical material candidate for graphene-based electronic devices.

In earlier works [7,8], several groups have fabricated pristine thin-film-GO-based field effect transistors and resistive switching memory devices. In these applications, GO acts as a charge transport layer. These works indicate that the direct current (DC) electrical transport properties of GO films is temperature-dependent, and GO film exhibits p-type semi-conducting characteristic at room temperature in ambient. Since GO contains *sp*^3^-bonded hybridized carbon atoms, it is worth noting that GO can capture water vapor from external environment easily, owing to its notable hydrophilicity [18-20]. Hence, studies on the effect of atmosphere relative humidity (RH) on the electrical and mechanical properties of GO are beneficial to the application of practical GO-based electrical device. Previous studies have noted that the water adsorption of GO can affect its structural and mechanical properties [18,19]. The uptake of water molecules increases the interlayer distance of GO sheets and forms hydrogen-bonding networks. In addition, an interesting phenomenon about the interaction of water vapor with GO films has been recently reported by Geim and his colleagues [20]. They have found that water molecules can readily permeate through GO films without blockage; however, other molecules including ethanol, hexane, acetone, decane and propanol don't show this characteristic. Until now, the effect of water adsorptions on electrical properties of GO is still undefined in physics. Thereby, the investigation of this effect is essential in the development of graphene electronics, especially graphene device originating from solution-processed GO. In this paper, we use both DC measurement and alternating current (AC) impedance spectroscopy methods to elucidate the effect of ambient humidity on the electrical properties of GO films.

## Methods

Graphite oxide was synthesized via the oxidative treatment of natural graphite using the modified Hummer's method [21]. Then, graphite oxide was exfoliated to single-layered GO sheets by ultrasonicating graphite oxide suspension for 1 h. The obtained brown suspension was used as coating solution. Atomic force microscope (CSPM5500, Benyuan, China) was used to characterize the apparent heights of the obtained GO sheets. Fourier-transform infrared (FT-IR) spectrometer (5700, Nicolet, USA) was used to characterize the FT-IR spectra of the GO film.

Interdigitated electrodes (IDEs) were fabricated on an n-type silicon wafer with a top layer of SiO_2_ (300 nm) formed by thermal oxidization. Ti/Au layers with the thickness of 100:400 nm were deposited on SiO_2_ layer using magnetron sputtering. The Au electrodes with a 20-μm-wide gap were formed through photolithography followed by wet etching. Before being functionalized by GO films, the IDE was rinsed with distilled water and ethanol and dried in vacuum overnight. The device was fabricated by dispersing the GO suspension onto Au IDE. A few drops (4 μl) of the GO suspension were cast onto Au IDE by a micro-syringe. After drying at room temperature for 6 h, a discrete network of GO sheets was left on the Au IDE.

**Figure 1:**
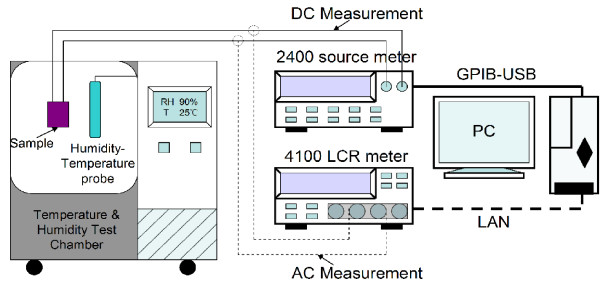
The experimental setup.

The schematic diagram of the experimental setup is shown in Figure 1. A temperature and humidity generator (LP-80U, Hongzhan, China) was used to generate the required humidity level at a fixed temperature. To evaluate the effect of atmosphere humidity on the electrical transport behaviors of GO films, the sample was placed in the chamber of the temperature and humidity generator. The DC measurements and AC complex impedance spectroscopy of the GO-film-functionalized IDE were performed by using a source measurement unit (Keithley 2400, Keithley Instruments Inc., USA) and an LCR meter (Wayne Kerr, 4100, Wayne Kerr Electronics, UK), respectively. All the experiments were carried out at a fixed temperature of 298 K.

## Results and discussion

### Characterizations of material and device

**Figure 2:**
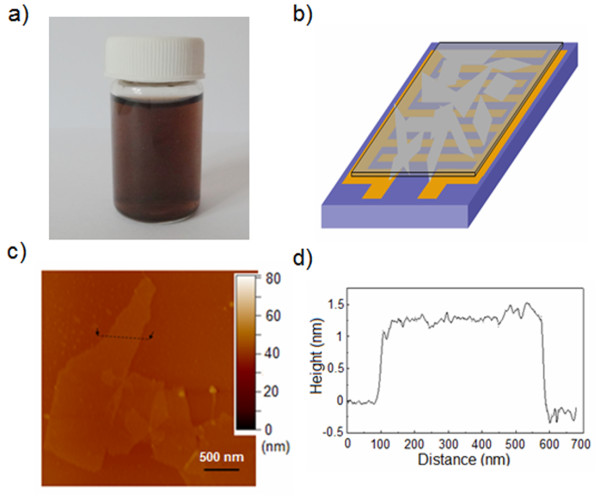
**GO-film-functionalized IDE.** (**a**) Photograph of stable GO dispersion (1 mg ml^−1^). (**b**) Schematic illustration of the IDE functionalized by GO films. (**c**) Tapping-mode AFM image of the GO sheets. (**d**) The height curve of the selected GO sheet shown in (c).

**Figure 3:**
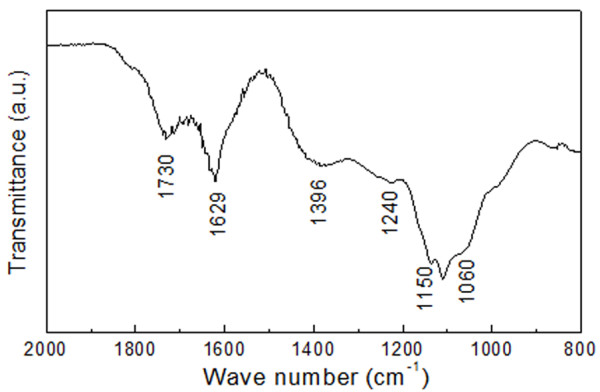
FT-IR spectra of the GO film.

The photograph of the stable GO dispersion (1 mg ml^−1^) is shown in Figure 2a, and Figure 2b illustrates the GO-film-functionalized IDE. Tapping-mode AFM image of the GO films in Figure 2c indicates that the GO sheets have a unique layered structure. The lateral size of a GO sheet ranges from several hundred nanometers to several micrometers. It is also observed from Figure 2c that there is a partial sheet-to-sheet overlap on the GO films. Figure 2d shows the typical apparent height of the observed single GO sheets. The thickness of the GO sheets is about 1.4 nm, which indicates that the GO sheets are predominantly single-layered. Figure 3 shows the FT-IR spectra of the GO film in the range of 2,000 to 1,000 cm^−1^. The peaks between 1,800 and 1,050 cm^−1^ are due to C = O, C–H, C–OH, C–O–C and C–O stretching at 1,730, 1,629, 1,396, 1,240 and 1,060 cm^−1^, respectively.

### DC electrical property of GO films at various humidity levels

The DC electrical property, i.e., current–voltage (*I-V*) characteristics of the GO-film-functionalized IDE, is measured with a voltage sweeping mode at various humidity points. In this configuration, one electrode of the IDE is loaded with the sweeping voltage bias, and the other electrode is grounded. Recent works have noted that the DC electrical property of GO films can be influenced by the amplitude of sweeping voltage [13,14]. Thereby, we investigate the *I-V* characteristics of GO films with low (−1 to 1 V) and high (−4 to 4 V) sweeping voltages at various humidity levels, respectively.

**Figure 4:**
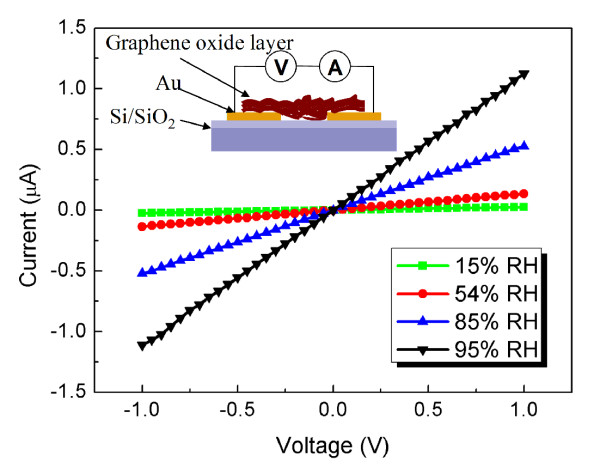
**Measured*I-V*characteristics of GO films in the humidity range of 15% to 95%.***I-V* characteristic of the GO-film-functionalized IDE with a low sweeping voltage (-1 to 1 V) at various RH levels. The inset figure shows the measurement configuration of the electrical connection.

Firstly, a sweeping voltage of −1 to 1 V is applied to the GO-film-functionalized IDE. Figure 4 shows the measured *I-V* characteristics of GO films in the humidity range of 15% to 95%. More clearly, the *I-V* curves of the GO-film-functionalized IDE are approximately linear for each humidity level; it indicates that well-ohmic contact formed between the electrode and the GO films. The reciprocal of the slope of the *I-V* curve represents the resistance of the GO films. The channel current of the GO-film-functionalized IDE increases with increasing RH, indicating that the water adsorption results in an increase in the resistance of GO films. For example, the amplitude of the resistance of the GO-film-functionalized IDE is approximately 10^8^ Ω at 15% RH, which is nearly ten times greater than that of GO films at 95% RH. The result shows that GO can be seen as an insulator in dry environment. However, a transition of GO from an insulator to a weak conductor occurs with a continuous water adsorption onto GO films.

**Figure 5:**
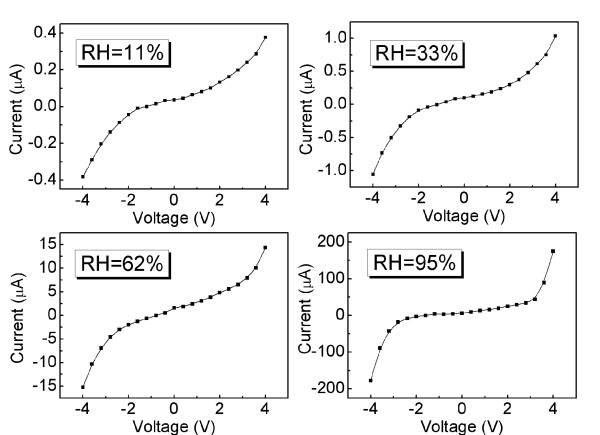
**Measured*I-V*characteristics of GO films applied with sweeping voltage of −4.0 to 4.0 V.***I-V* characteristic of the GO-film-functionalized IDE with high sweeping voltage (-4 to 4 V) at various RH levels.

Next, a sweeping voltage of −4.0 to 4.0 V has also been applied to the GO-film-functionalized IDE. The *I-V* curves of the device at various humidity levels are shown in Figure 5. It can be found that all the curves exhibit similar variation tendencies. In the voltage range of −2.0 to 2.0 V, the *I-V* curves are approximately linear; meanwhile, the channel current of the device increases with increasing RH. This result is in agreement with the *I-V* characteristics under a low sweeping voltage shown in Figure 4. When the sweeping voltage extends beyond 2 V, the channel current increases with increasing the amplitude of the sweeping voltage; it indicates that the conductivity of GO films increases with increasing the sweeping voltage. In addition, we can also find that the channel current under high sweeping voltage increases with increasing humidity level. For instance, the resistances at 11%, 33%, 62% and 95% RH under the sweeping voltage of 4 V are 10.6, 3.9, 0.278 and 0.023 MΩ, respectively.

The mechanism of electric-field-induced reduction of GO films is used to explain the observed phenomenon above [13,14]. When a high sweeping voltage is applied on the device, the exciting electric field will be in interaction with the GO films. The ionization of interlayer water molecules in the GO films leads to yields of hydrogen ions and hydroxyl ions due to the strong electric field. Then, the reduction of GO to reduced GO (rGO) occurs according to Equation 1 [13]. Meanwhile, this reduction process is reversible within a short sweeping time (sweeping delay time of 1 s in this work) according Teoh's result [13]. It should be noted that the water content of GO films plays an important role in this reduction process. When ambient RH is high, the number of adsorbed water molecules is large. As a result, the ionization process generates more hydrogen ions, which are involved in the reduction process of GO films. Thereby, GO films exhibited high conductivity in the case of a high applied voltage at a high humidity level.

(1) $$\text{GO}+2{\text{H}}^{+}+2e^{-}=>rGO+{\text{H}}_{2}\text{O}$$

### AC complex impedance spectroscopy of GO films at various humidity points

**Figure 6:**
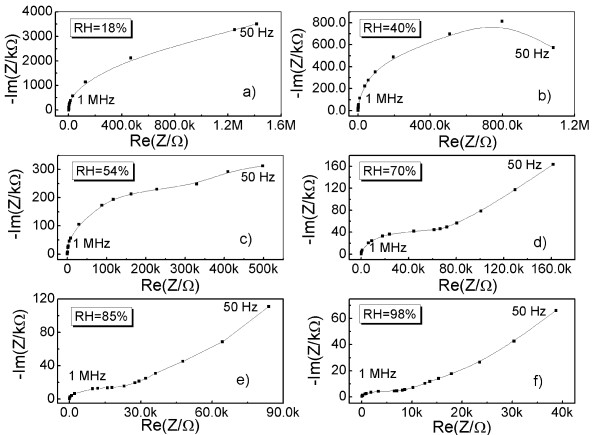
**Measured complex impedance spectroscopy of the GO-film-functionalized IDE.** Complex impedance spectroscopy using a Cole-Cole representation at (**a**) 18%, (**b**) 40%, (**c**) 54%, (**d**) 70%, (**e**) 85% and (**f**) 98% RH, repectively.

**Figure 7:**
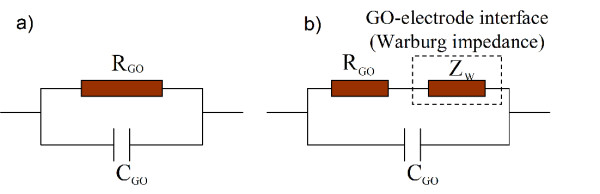
**Equivalent circuits of the GO-film-functionalized IDE.** (**a**) Semicircle-type impedance associated with the bulk impedance of GO films and (**b**) both semicircle-type impedance associated with the bulk impedance of GO films and straight line-type impedance (i.e., Warburg impedance) associated with GO-electrode interface.

It is well known that AC complex impedance spectroscopy (Nyquist plot of impedance) provides a powerful tool to analyze the electrical interaction of the investigated material with water molecules [22-24]. To know the electrical interaction of GO films with water molecules in detail, the measurements of AC complex impedance spectroscopy are carried out in the frequency ranging from 50 Hz to 1 MHz. The amplitude of the exciting signal is 500 mV. Figure 6 shows the measured complex impedance spectroscopy using a Cole-Cole representation at various RH levels. It can be found that these Cole-Cole plots exhibit two types of impedance: semicircle-type impedance and straight line-type impedance. Regarding the two types of impedance, many researchers have provided an interpretation using dielectric physics theory [22,23]. The semicircle-type impedance is associated with the bulk impedance of GO films, and it can be electrically equivalent to a parallel circuit of a resistor *R*_GO_ and a capacitor *C*_GO_, as shown in Figure 7a where *R*_GO_ and *C*_GO_ represent the bulk resistor and geometric capacitance of the GO films, respectively. The straight line-type impedance named Warburg impedance was caused by the diffusion of ions across the interface between the GO films and the electrodes; it can be modeled as additional impedance *Z*_w_, as shown in Figure 7b [23].

**Figure 8:**
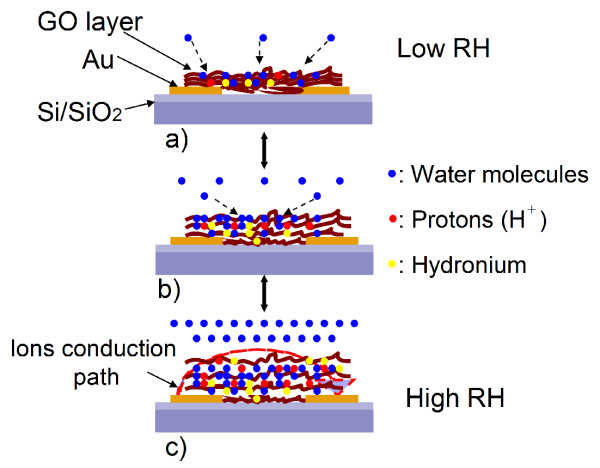
**The electrical interaction mechanism between GO films and water molecules.** At (**a**) low RH, (**b**) medium RH and (**c**) high RH.

At lower RH (<54% RH), only semicircle-type impedance is observed in Figure 6a,b. For GO-film-functionalized IDE, chemisorption occurred at the beginning (water molecules act as a donor, which combines adsorbed oxygen at the surface of the GO films to form hydroxyl) and then followed by physisorption. As described by Anderson's proton conductivity model [25], at a low RH, only a small amount of water molecules is absorbed onto the surface active hydrophilic groups (i.e., hydroxyl) of GO sheets through hydrogen bonding, and hence, the GO surface is not completely water-covered. With the increasing adsorption of water molecules, hopping proton mechanism plays an important role. Protons (H^+^) arising from hydroxyl of GO is bonded to excess adsorbed water molecules to form hydronium (H_3_O^+^) ion. However, the hydronium ions formed by charged carriers were alone and not enough to yield the continuous conduction path due to insufficient adsorption of water molecules. Thus, this process does not bring out conductance obviously. However, the increasing formation of hydronium ions can be seen as an accumulate layer. The mechanism is illustrated by a cartoon image shown in Figure 8a. At the same time, the polarization occurred between the GO films and the electrodes due to applied electrical fields and produced bounded electrons. Thereby, we can consider that the complex impedance was mainly contributed by the intrinsic impedance of GO films at low RH. In this case, the total impedance is composed of the bulk resistor *R*_GO_ and geometric capacitance *C*_GO_ of the GO films, as shown in Figure 7a. In agreement with the DC measurement discussed above, a larger diameter of semicircle observed in the Cole-Cole plot is due to the inherent poor conductivity property of GO films.

As the humidity increased stepwise above 54% RH, straight line-type impedance appears at the low-frequency region, and semicircle-type impedance appears at the high-frequency region shown in Figure 6c,d,e,f. In this case, water vapor concentration reaches a higher value. More and more water molecules are absorbed onto the surface of GO films, resulting in an increase of hydronium ions through the hopping mechanism mentioned above. As a result, the bulk conductivity of GO films increases with increasing RH, resulting in a decrease of the diameter of the semicircle. Meanwhile, partial hydronium ions are hydrated into water molecules and H^+^ afresh, leading to a formation of a liquid layer around the interlayer of GO sheets by two-dimensional capillary or swelling effect. This process increases the interlayer distance of GO sheets largely, which can be sufficient to accommodate a monolayer of water. The formation of a liquid layer provides a conduction path across, between GO films and electrodes as illustrated in Figure 8c, increasing the mobility of the diffusion ions (including hydronium ions and H^+^). As a result, ion conduction, i.e., Warburg impedance *Z*_w_, appears. Therefore, Warburg impedance *Z*_w_ is added in the equivalent circuit shown in Figure 7b. When RH increased above 80% RH, the Warburg impedance became dominant. Thus, we can consider that the major conduction process is attributed to adsorbed-water-induced ion conduction at high RH rather than the intrinsic conductivity of GO films. Based on the discussion above, we summarized the interaction mechanism of GO films with different amounts of water molecules, which was illustrated in Figure 8.

### The dependence of the exciting frequency on impedance versus humidity response of GO films

**Figure 9:**
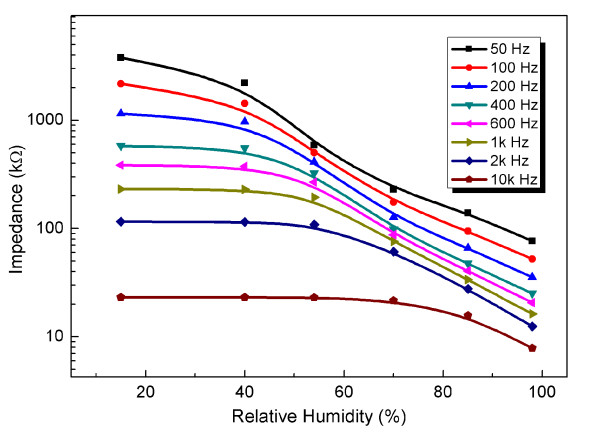
The dependence of the exciting frequency on impedance versus humidity response of GO films.

Figure 9 plots the measured impedance magnitude as a function of humidity and frequency. The results display the following features. The amplitude of impedance exhibits a decrease with increasing RH for all investigated frequency points. Moreover, the response amplitude of impedance versus RH shows frequency dependency. At low test frequency points (below 1 kHz), the amplitude of impedance exhibited an entire decrement with the increase in RH. At high test frequency points (beyond 1 kHz), the amplitude of impedance was initially insensitive to humidity below 54% RH and showed a gradual decrease above 54% RH. This behavior can be interpreted by molecule dielectric physics. At high frequencies, the applied electrical field altered rapidly; as a result, the ions arising from adsorbed water molecules cannot catch up with the alternating rate of the applied electric field due to its large relaxation time [26]. Hence, the dielectric property was weak and showed insensitivity to RH. In Figure 9, the amplitude of impedance decreases from the order of megaohm to several hundreds kilohm at the test frequency of 50 Hz when humidity varied from 18% to 98% RH. This result suggests that GO films can be used as an impedance-type humidity sensor at a certain frequency range.

## Conclusions

We used the DC measurement and AC complex impedance spectroscopy methods to investigate the effect of ambient humidity on the electrical properties of GO films. The strong interaction of water molecules with GO films was observed through electrical characterizations. The DC measurement results show that the electrical properties of GO films were affected by ambient humidity and the amplitude of applied voltage. The electrical sensing mechanism of GO films was discussed by analyzing the characteristics of AC complex impedance spectroscopy. At low RH (<54%), GO films exhibited poor conduction property due to the presence of *sp*^3^-bonded hybridized carbon atoms. As RH increased stepwise above 54%, the conductivity of GO films increased sharply due to strong water-adsorption-induced ion conduction. The results are beneficial to the development of graphene-based electronics, especially graphene device arising from solution-processed GO. Finally, the exciting-frequency-dependent impedance of the GO films versus humidity was discussed, and this result suggested a potential application of GO films in impedance-type humidity sensor.

## Competing interests

The authors declare that they have no competing interests.

## Authors’ contributions

The experiment and initial manuscript were prepared by YY and XDC. XYL helped in the AFM scan. ZQW helped in the FT-IR analysis. BQZ and JFZ contributed valuable idea and useful discussion for this manuscript. All authors read and approved the final manuscript.
